# KCTU randomisation and IMP management system

**DOI:** 10.1186/1745-6215-14-S1-P63

**Published:** 2013-11-29

**Authors:** Caroline Murphy, Joanna Kelly, John Hodsoll, Andrew Pickles, Evangelos Georgiou, PL Morgan

**Affiliations:** 1 King's Clinical Trials Unit at KHP, Institute of Psychiatry, King's College London, London, UK; 2 Biomedical Research Centre for Mental Health, South London and Maudsley NHS Trust, London, UK

## 

The management of medicinal clinical trials commonly presents challenges to trial teams, particularly when seeking practical and affordable systems for managing the trial randomisation process and associated Investigational Medicinal Product (IMP) distribution.

In blinded IMP trials, these challenges include:

• 1) how to manage IMP supply when the preferred randomisation algorithm does not guarantee balance between trial arms in each centre

• 2) tracking of IMP expiry dates and stock levels to ensure continuous supply of both trial arms at site

• 3) tracking availability of central stock of manufactured IMP to facilitate planning of subsequent manufacturing runs

To provide an efficient and robust solution to these challenges, a web-based randomisation system with integrated IMP management, is presented.

Designed within the King's Clinical Trials Unit, the system accommodates simple randomisation, block randomisation, stratified block randomisation and minimisation, enabling trial teams to manage blinded IMP distribution to sites while remaining fully blinded to treatment allocation. Blinding is maintained even where there is gross imbalance between trial arms of patients within a study site.

This system avoids the need for IMP manufacturing companies to take on this role. Further, it avoids the need for individuals within the study management team to be unblinded while managing the IMP supply during the conduct phase. It has also been designed to accommodate many of the common events that occur in clinical trials, such as patients losing packs of medication or IMP expiry dates being extended mid-study.

